# Lipid-Based Delivery Systems for Flavonoids and Flavonolignans: Liposomes, Nanoemulsions, and Solid Lipid Nanoparticles

**DOI:** 10.3390/pharmaceutics15071944

**Published:** 2023-07-14

**Authors:** Shahla Ranjbar, Abbasali Emamjomeh, Fatemeh Sharifi, Atefeh Zarepour, Kian Aghaabbasi, Ali Dehshahri, Azadeh Mohammadi Sepahvand, Ali Zarrabi, Hamid Beyzaei, Mohammad Mehdi Zahedi, Reza Mohammadinejad

**Affiliations:** 1 Department of Plant Breeding and Biotechnology, Faculty of Agriculture, University of Zabol, Zabol 9861335856, Iran; 2 Research Center of Tropical and Infectious Diseases, Kerman University of Medical Sciences, Kerman 7616913555, Iran; 3 Department of Biomedical Engineering, Faculty of Engineering and Natural Sciences, Istinye University, 34396 Istanbul, Turkey; 4 Department of Biotechnology, University of Guilan, University Campus 2, Khalij Fars Highway 5th km of Ghazvin Road, Rasht 4199613776, Iran; 5 Department of Pharmaceutical Biotechnology, School of Pharmacy, Shiraz University of Medical Sciences, Shiraz 7146864685, Iran; 6 Pharmaceutical Sciences Research Center, Shiraz University of Medical Sciences, Shiraz 7148664685, Iran; 7 Department of Chemistry, Faculty of Science, University of Zabol, Zabol 9861335856, Iran; 8 Department of Chemistry, University of Saskatchewan, 110 Science Place, Saskatoon, SK S7N 5C9, Canada

**Keywords:** bioavailability, lipid-based nanocarrier, flavonoids, flavonolignans, nanocarrier

## Abstract

Herbal chemicals with a long history in medicine have attracted a lot of attention. Flavonolignans and flavonoids are considered as two classes of the above-mentioned compounds with different functional groups which exhibit several therapeutic capabilities such as antimicrobial, anti-inflammatory, antioxidant, antidiabetic, and anticancer activities. Based on the studies, high hydrophobic properties of the aforementioned compounds limit their bioavailability inside the human body and restrict their wide application. Nanoscale formulations such as solid lipid nanoparticles, liposomes, and other types of lipid-based delivery systems have been introduced to overcome the above-mentioned challenges. This approach allows the aforementioned hydrophobic therapeutic compounds to be encapsulated between hydrophobic structures, resulting in improving their bioavailability. The above-mentioned enhanced delivery system improves delivery to the targeted sites and reduces the daily required dosage. Lowering the required daily dose improves the performance of the drug by diminishing its side effects on non-targeted tissues. The present study aims to highlight the recent improvements in implementing lipid-based nanocarriers to deliver flavonolignans and flavonoids.

## 1. Introduction

**Figure 1:**
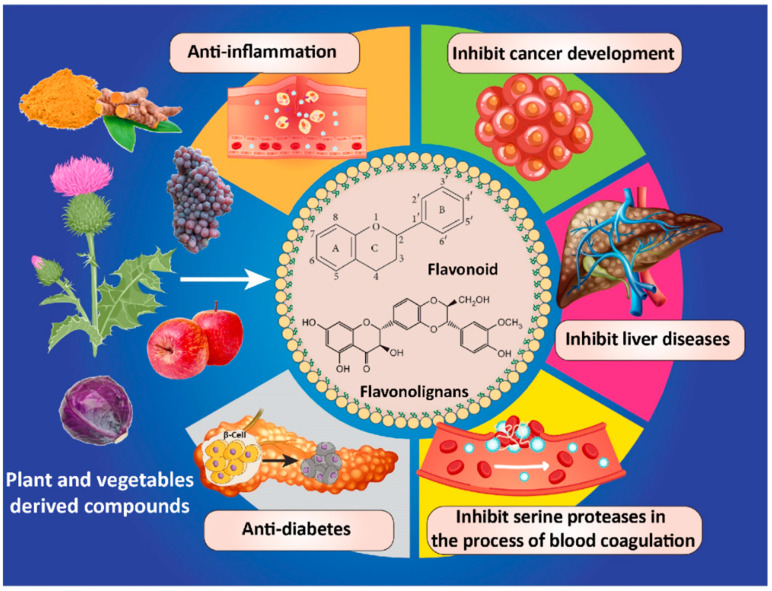
Different biological functions of flavonoids and flavonolignans.

Plants are among the primary sources of pharmacologically active substances used to treat illnesses throughout human history, resulting in a branch of medicine emerging known as herbal medicine [1,2,3]. Based on the studies, two phenolic compounds, flavonoids and flavonolignans, contain C6-C3-C6 carbon skeletons. Flavonolignans contain two components, flavonoid and phenylpropane. Flavonoids are regarded as a superfamily of polyphenols containing seven different subclasses: flavones (tangeretin, luteolin, aspigenin, etc.), flavonols (quercetin (Que), kaempferol, myricetin, rutin, etc.), flavanones (hesperidin, hesperetin, naringenin, etc.), isoflavonoids (genistein, daidzein, etc.), anthocyanidins (delphinidin, pelargonidin, cyanidin, malvidin, etc.), flavan-3-ols (epigallocatechin, theaflavins, catechin, epicatechin gallate, proanthocyanidins, epigallocatechin gallate, etc.), and chalcones (flavokawain C, calomelanone, butein, homobutein, 4-hydroxychalcone, isoliquiritigenin, etc.) [4,5,6,7,8]. The aforementioned cases are considered as secondary metabolites which can be found in different parts of plants from flowers and fruits to roots, barks, grains, and stems. The above-mentioned classes of polyphenols exhibit excellent anti-mutagenic, anti-cancer, antibacterial, antioxidant, antiviral, antifungal, and anti-inflammatory behavior [9,10] which makes them potential candidates for treating various types of cancer and neurodegenerative diseases, diabetes, cardiovascular disease, and wound healing (Figure 1) [11,12,13].

Flavonolignans are regarded as a small subgroup of polyphenols first found in silymarin (SLM), the seed extract of the Silybum marianum, which contains different flavonolignans, silychristin (SCN), silybin (SBN), silydianin (SDN), and isosilybin (ISBN) [14]. Typically, flavonolignan is utilized to treat liver diseases including alcoholic liver diseases, hepatitis, and liver cirrhosis [15,16,17]. Other applications of the flavonolignan family include but are not limited to anti-inflammatory, anti-cancer, immunomodulating, antioxidant and, antifibrotic compounds, as well as ameliorating chemotherapy side effects (Figure 1) [16,18].

The widespread application of the aforementioned families is constrained by a number of obstacles such as poor water solubility, low membrane and intestinal permeability, low stability, easy degradation under strong acidic conditions, easy metabolism, and quick removal from the blood via immune system and renal clearance, despite their advantages [19,20,21,22,23,24].

As shown, different formulations have been created to enhance the bioavailability of the above-mentioned compounds [25,26], the most significant of which is applying nanomaterials as shielded delivery vehicles [12,27,28,29,30]. Nanotechnology and nanoscience represent novel and promising platforms to provide an extensive range of new applications and enhanced technologies in different fields due to recent improvements. This is especially true for environmental, biological, and biomedical applications [31,32,33,34,35,36,37,38]. Based on the reports, using nanomaterials as drug delivery exhibits numerous advantages such as targeted delivery, increasing intracellular retention time, improving bioavailability, reducing side-effects, and controlling release patterns [39,40,41,42].

Thus, proper selection of formulation techniques and compositional materials in designing therapeutic delivery systems plays a crucial role in producing a marketable, safe, and functional product [43]. Lipid-based carriers such as solid lipid nanoparticles (SLNs) [44,45], micro/nanoemulsions [46,47], and liposomes [48,49,50] are among the key examples of successful carriers in solubilizing flavonoids and flavonolignans in aqueous environments. This study seeks to assess recent developments in formulation tactics which have made flavonoids and flavonolignans more biologically available. Here, lipid-based nano delivery systems and their application towards flavonoids and flavonolignans in pharmaceuticals are discussed. Finally, the clinical applications, limitations, and potential future applications of lipid-based nano delivery systems are elaborated more.

## 2. Lipid-Based Carrier Systems

Drug failure occurs in vivo due to poor absorption and/or distribution, high toxicity, low solubility, and unpredicted bioavailability, as well as poor drug metabolism [51]. Controlled and localized drug release plays a critical role in overcoming such drawbacks utilizing an appropriate drug carrier system. Lipid-based components are considered as promising materials for such delivery systems [52,53,54,55,56].

Physicochemical properties of lipids such as biocompatibility, low susceptibility to erosion phenomena, and slow water uptake make lipids an ideal nanocarrier system to improve active pharmaceutical ingredient (API) aqueous solubility, bioavailability, and effective therapy. In addition, lipid-based systems improve API storage and delivery while inhibiting API oxidation, degradation, and decomposition [57]. Unlike other delivery systems, lipid-based drug delivery systems exhibit a major advantage over other methods due to their ability to cross the gut, gastro-intestinal tract (GIT), blood vessels, and blood brain barrier [58,59]. Some lipid-based drug delivery systems include self-emulsifying systems [60,61], SLNs [62,63], nanostructured lipid carriers (NLCs) [64,65], and liposomes [66].

### 2.1. Liposomes

Liposomes are regarded as the most widely applied technology for drug delivery on the market. This drug delivery system is called a lipid bilayer or a phospholipid vesicle. Liposomes have attracted a lot of attention due to their easy preparation methods and high drug loading efficiency. Further, liposomes have slow and targeted release of payloads [67]. The term liposome defines a mesomorphic structure made of lipid, phospholipid, and water. Liposomes mainly contain phospholipid molecules, which entrap and release lipid-soluble, amphiphilic materials, as well as water-soluble compounds, in a controlled manner to enhance the efficacy of nutraceuticals, pharmaceuticals, and other bioactive compounds [68]. Liposomes have been used in the pharmaceutical industry since 1995 thanks to US Food and Drug Administration (FDA) approval [69].

Generally, liposomes include closed phospholipid double layers and benefit from a hydrophilic core unlike micelles. Liposomes may carry amphiphilic, hydrophobic, and hydrophilic compounds [70,71,72,73]. In other words, the polar substance is loaded into the hydrophilic core, while the hydrophobic compounds may be placed inside the bilayers of lipid domains [73,74]. The term nanoliposome is derived from ‘liposome’ (lipos: fat and soma: body), meaning lipidic structures with nanoscale dimensions. Nanoliposome exclusively indicates lipid vesicles at nanoscale, while liposome is considered as a general term utilized to cover different groups of lipid vesicles on the diameters ranging between tens of nanometers to several micrometers [75].

Liposomes and nanoliposomes are categorized based on diameter range, number of internalized vesicles, and number of lamellas. Lipid vesicles may be prepared as a small unilammelar vesicles (SUVs) which are created by a bilayer membrane. Large unilammelar vesicles (LUVs) with approximately 20–100 nm diameter range benefit from a bilayer membrane, while double bilayer vesicles (DBVs) contain two bilayer membranes with above 300 nm diameter range. Oligolammelar vesicles (OLVs) contain 3–5 bilayers, while multilammelar vesicles (MLVs) contain more than five concentric bilayer vesicles. There are giant unilammelar vesicles (GUVs) which are generated by a bilayer, as well. Multivesicular vesicles (MVVs) include a bilayer liposome which encapsulates numerous small non-concentric vesicles. Nanoliposomes or sub-micron lipid vesicles are found as SUV, LUV, DBV, and OLV, while MVV, GUV, MLV, OLV, DBV, and LUV are regarded as liposomes [75,76]. There are numerous methods to create liposomes including reverse-phase evaporation (RPE) [77], thin film dispersion (TFD) [78], spray-freeze-drying [79], and alcohol injection [80,81]. More environmentally friendly methods involve pro-liposomal formulation applying a cryoprotectant and high-temperature procedure, as well as supercritical fluid of carbon dioxide (SCF-CO2) techniques to adjust the experimental pressure and temperature as a method to control the shape and size of the particles [47].

#### 2.1.1. Flavonoid Liposomes

**Figure 2:**
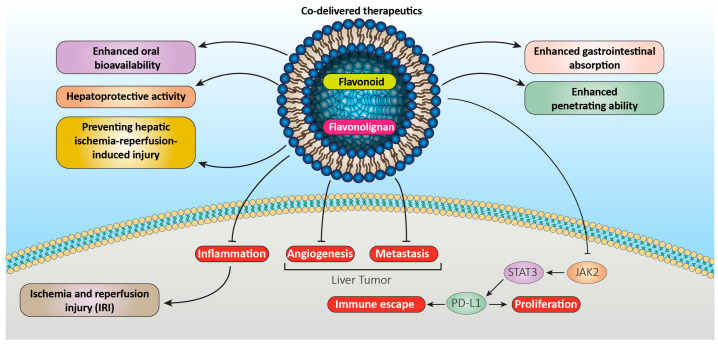
Liposomal formulations of flavonoids and flavonolignans improve their therapeutic efficacy.

According to Jing et al., folic-acid-modified liposomes improved quercetin anti-tumor properties significantly. With this aim, a novel method of treating osteosarcoma was displayed by proving that quercetin inhibits the JAK2-STAT3-PD-L1 signaling axis role in the immune response to osteosarcoma [82]. In addition, Tang et al. [83] evaluated the impact of quercetin liposomes (Q-PEGL) on rats with streptozotocin (STZ)-induced diabetic nephropathy (DN) and used the technique for intragastric injection drug administration. The results indicated that quercetin-loaded liposomes could be successfully generated from proper formulation of quercetin, lecithin, cholesterol, and polyethylene glycol 4000. An animal study revealed that the formulated quercetin-loaded liposomes can demonstrate kidney-protective properties in rats with STZ-induced DN, reduce DN progression rate, and decrease AGE expression. Quercetin liposomes impact DN more than quercetin alone and can be utilized as an effective treatment for DN [83]. A stable quercetin liposomal formulation with anti-inflammatory and antioxidant activities was prepared and studied by Ferreira-Silva et al. for treating hepatic ischemia and reperfusion injury (IRI). The results indicated that implementing quercetin in liposomal nano carriers increased therapeutic impact both in vitro and in vivo, supporting the possibility of such approach for hepatic IRI treatment [84]. In another study, Li et al. [85] encapsulated quercetin in the PEGylated liposomes’ non-aqueous interior and indicated that quercetin-loaded PEGylated liposomes (PEG-Que-NLs) exhibit promising anticancer effects in vitro and in vivo due to the improved solubility and bioavailability of natural quercetin. Based on the results, PEG-Que-NLs may target tumors, release medication gradually, and increase quercetin solubility. Therefore, PEG-Que-NLs may be applied in treating malignant tumors [85]. Further, Zhang et al. [86] proposed employing the quercetin liposome as a potential allergy antagonist to elucidate the anti-allergic action of quercetin liposomes on RBL-2H3 cells in vitro. The results indicated that quercetin liposomes can reduce the release of histamine and beta hexosaminidase, calcium influx, and expression of inflammatory markers significantly compared to quercetin alone [86]. Furthermore, Li et al. examined whether liposomal quercetin (LQ) may improve the outcomes of microwave ablation (MVA) while treating the rabbit VX2 liver tumor model. The results indicated that the preparative infusion of LQ can significantly improve the effects of MWA on VX2 liver tumors in rabbits, extend their survival time by inhibiting the expression of HSP70 and HIF-1 in the residual tumor, and prevent the excessive growth of the residual tumor by lowering metastasis (Figure 2) [87].

Renault-Mahieux et al. proposed the idea of co-encapsulating fisetin and cisplatin into liposomes to combine the antiangiogenic properties of fisetin with the cytotoxic properties of cisplatin. Based on the results, the fisetin antiangiogenic properties remained intact, encapsulated cisplatin and fisetin affected glioblastoma (GBM) cells, and fisetin impacted GBM U87-MG cells. In other words, the co-loaded formulation exhibited effective anti-GBM cell activity and could keep fisetin effects long-lasting [88]. In addition, Altamimi et al. reviewed the efficacy of transdermal elastic liposomes (LEL1-LEL12) loaded with luteolin (LUT) to prevent breast cancer in vitro and ex vivo. The drug-loaded carrier (OLEL1) displayed concentration-dependent inhibition of MCF-7 cells compared to the free drug and the elastic liposome improved cellular internalization for maximum inhibition, resulting in establishing a potential strategy of elastic liposomes for improving LUT transdermal administration and increasing therapeutic effectiveness in breast cancer treatment [89]. Further, Deshmukh et al. created chrysin liposomes (CLPs) using the chitosan/lecithin/electrostatic deposition assisted film hydration approach to protect chrysin in the nano-lipoidal shell. A more than five-fold increase in chrysin liposome bioavailability was observed in in vivo pharmacokinetic research. In silico molecular docking studies of the same combination reported electrostatic interaction between the two polymers, indicating that chitosan might shield and enclose chrysin, resulting in increasing its cytotoxicity and bioavailability in the process [90]. Furthermore, Huang et al. created a liposomal chrysin (LC) and discussed its effects on hepatic ischemia-reperfusion (HIR) and potential mechanisms. The results indicated that LC benefits from promising biocompatibility and is considered as an effective medication for treating and preventing HIR-induced injury [91]. In another study, Tian et al. prepared PSMA-specific antibodies co-encapsulated in liposomes with genistein and plumbagin to target prostate cancer cells. The above-mentioned liposomes reduced the development of PSMA-expressing prostate cancer cells by more than 90% without harming healthy cells or human red blood cells. Overall, encapsulating other treatments utilizing the same method can create non-toxic and PSMA-specific antibody linked liposomes such as the drugs genistein and plumbagin to improve various cancer treatments and stop their spread [92].

#### 2.1.2. Flavonolignan Liposomes

In fact, one of the first SLM liposomal formulations was reported during the early 2000s. A number of factors including the drug-to-lipid ratio were discussed for preparing the aforementioned formulations [50]. The formulation cholesterol ratio and inclusion of dicetylphosphate (DCP) as a charge inducer were optimized by injecting ethanol. A ratio of 10:2:2:1 for L-α-phosphatidylcholine (PC), SLM, cholesterol (CHOL), and DCP was applied to achieve 95% drug entrapment. The SLM-loaded liposomes benefited from a medium diameter of 390 nm and a size range of 56–1270 nm, which are regarded as appropriate for intravenous delivery during hepatoprotective tests in mice.

Ochi et al. described a recent development in the use of ligand-functionalized liposomes to increase SLM bioavailability [93]. With this aim, a synergic effect on HepG2 cells was established by co-entrapment of glycyrrhizic acid (GA) and SLM into PEGylated liposomes. Liposomes prepared by the TFD method were formulated employing GA and SLM at a molar ratio of 1:1.74 combined with mPEG2000-DSPE, CHOL, and dipalmitoylphosphatidylcholine (DPPC) at a constant molar ratio. The results indicated that the co-encapsulated liposomes had an average diameter of about 43 nm and a zeta potential of −23.25 mV, which prevents liposome aggregation. The cellular uptake and antiviral activity of SLM encapsulated in phytoliposomes were analyzed in vitro against Huh-7.5 cells [94]. The results indicated a 2.4-fold cell absorption compared to free SLM, as well as 300-fold more potent pharmacological activity.

Methods using the SCF-CO2 technique were utilized to create liposomes through SLM inside. In particular, liposomes applying sodium glycocholate (SGC) and soya hydrogenated L-α-phosphatidylcholine (HSPC) as the basis of lipid materials were prepared employing Solution-Enhanced Dispersion Supercritical (SEDS) fluids [95]. The optimized products displayed improved anti-inflammatory action after comparing the liposomes created by the optimized products to those generated with more traditional techniques such as RPE and TFD. According to an in vitro analysis of the release profiles, the updated formulation enhanced SLM solubility. Based on in vivo tests, oral bioavailability of the drug improved compared to the commercial product or aqueous drug suspension. Based on the studies, bile salt stabilized vesicles (bilosomes) and SLM contents affect the drug entrapment efficiency (EE) of bilosomes created by the TFD method. Comparing the achieved liposome dispersions to the equivalents created using CHOL rather than bile salts indicated that such dispersions were classified utilizing negatively charged values. The assessed bilosomes containing sodium cholate (SC) exhibited the largest nanoparticles (NPs) in terms of particle size (595.1–98.48 nm) [96].

Lecithin-pluronic organogels generated a novel nanocarrier for SLM in treating atopic dermatitis (AD) due to their adaptability and biphasic composition as transdermal and daily defense [97]. The evaluated formulations contained 80% aqueous phase (pluronic) and 20% oil phase isopropyl myristate (IPM/lecithin). The symptoms and signs of patients with AD improved significantly due to the organogel hydration effect and high penetration ability. Table 1 indicates some of the liposomal structures applied to encapsulate flavonoids and flavonolignans.

### 2.2. Micro- and Nanoemulsions

Micro- and nanoemulsions (NEs) have been extensively studied for their ability to encapsulate a wide range of lipophilic drugs. Although the terminology may differ, both microemulsion (ME) and NE oil droplets can have sizes in the nanometer range and their size distributions may overlap. However, there is no universally accepted definition of the nanometric range, as various authors have defined critical size values with upper limits of 100 nm, 200 nm, or 500 nm [99]. MEs contain water, oil, and surfactants which are thermodynamically stable. The small size of the droplets and surfactants contributes to the high stability and solubility of MEs, which makes them ideal for delivering drugs with low solubility. Although generally the same ingredients (oil, water, and surfactant) are required to produce NEs and MEs, the ratios of NEs and MEs may vary [100]. MEs require a higher surfactant to oil ratio (SOR) than NEs. MEs and NEs are considered as stable and unstable, respectively, in terms of thermodynamic stability [99]. The formation and stability of formulations is strongly influenced by the electrical properties of the surfactants. The surfactants can be classified into nonionic, zwitterionic, and cationic or anionic based on their electrical charge [101,102,103].

#### 2.2.1. Flavonoid Nanoemulsions

According to Shadab et al., a naringenin-loaded NE could be a potential therapeutic agent for Alzheimer disease. The direct neurotoxic effects of beta amyloid (Aβ) were reduced by a naringenin-loaded NE on SH-SY5Y cells, which was accompanied by a downregulation of amyloid precursor protein (APP), β-secretase expression. Such observations suggested ample reduction in amyloidogenesis, as well as decreased phosphorylated tau levels in human neuroblastoma cell line (SH-SY5Y) cells [104].

Kaplan (2019) created daidzein (DZ)-containing NEs and NE-based gels (NEGs) to test their cytotoxic potential while employed topically to treat melanoma. ProtasanTM UP G 213 (1% *w/w*) was added to the NE formulation after being created using a high-pressure homogenization process to generate NEG formulations. NE and NEG formulations with a controlled release profile and nanoscale droplet size were demonstrated for the topical use of DZ to treat melanoma [105]. In addition, Hussein et al. reduced cardiac toxicity and DNA damage by administering quercetin nanoemulsion (Que-NE) to an experimental diabetes model. Based on the results, turning QCT into a NE increased its solubility. The nano-delivery method shielded diabetic rats from cardiac toxicity and DNA damage through injecting QCT which is regarded as an anti-inflammatory and antioxidant drug [106]. Further, Mahadev et al. examined Que-NE with ultrasonically assisted dispersion for improving bioavailability and therapeutic efficacy against diabetes mellitus among rats. A Que-NE was created utilizing EO, T20, and labrasol as the appropriate oils, surfactants, and cosurfactants. The ultrasonically assisted Que-increased NE oral bioavailability raised quercetin therapeutic and preventive anti-diabetic properties significantly [107]. Furthermore, Ceramella et al. investigated the simultaneous encapsulation of quercetin and cisplatin to simplify drug administration. Two human cell models, normal HEK-293 kidney cells and human breast carcinoma MDA-MB-231, were applied to characterize and test the derived formulations. Employing NEs containing the above-mentioned substances lessened the principal cytotoxic activity of cisplatin on HEK-293 cells significantly. In addition, the antioxidant properties of encapsulated quercetin were enhanced [108].

In another study, Son et al. (2018) reviewed the hypocholesterolemic effects of a nanosized quercetin emulsion among rats fed with a high-cholesterol diet and characterized the physicochemical features of a double-layer oil-in-water NE encapsulated with quercetin (NQ) over a broad range of pH and various storage times. Complexation and self-assembly with Captex^®^ 355, Tween 80, sodium alginate, and soy lecithin were used to create a quercetin-loaded double-layer oil-in-water NE. NQ exhibited more promising outcomes than quercetin alone in decreasing blood and hepatic cholesterol levels. In addition, NQ enhanced the release of bile acid into stool among rats fed a high-cholesterol diet. Genes involved in bile acid synthesis and cholesterol efflux such as ATP-binding cassette transporter A1 (ABCA1), liver X receptor alpha (LXR), cholesterol 7 alpha-hydroxylase (CYP7A1), and ATP-binding cassette sub-family G member 1 were among those whose expression was studied by NQ (ABCG1) [109].

Ahmadi Oskooei created quercetin nanoemulsions (QuNEs) to improve quercetin solubility in polar aqueous environments and discussed the creation of quercetin–proteins (QuNE–human serum albumin (HSA) and QuNE–holo-transferrin (HTF)). QuNE exhibited substantial antioxidant benefits due to its ability to transport quercetin to HSA and HTF proteins and stabilize their protein complexes. The synthesis of stable and bio-accessible QuNE–proteins such as QuNE–human serum albumin (HSA) and QuNE–holo-transferrin (HTF) utilizing QuNE as an appropriate primary transporter led to a secure secondary delivery system with the potential to be exploited as an effective anticancer drug [110].

In addition, Marques analyzed the effects of quercetin and its NE on MDR and non-MDR cells applying high-pressure homogenization to make NEs. The effects of quercetin and vincristine equaled verapamil which is considered as an ABCB1 inhibitor. Docking argued that the aforementioned compounds bind to ABCB1 at a similar location. Such NE enhanced the bioavailability of quercetin by maintaining its cytotoxic and cytostatic properties. The NE ability to immediately stop ABCB1 efflux activity indicated the existence of a structure–activity link [111].

Further, Magura (2022) assessed hesperidin NE encapsulation and compared the cytotoxicity of the optimized hesperidin-loaded NEs (HPNEM) to hesperidin alone in MCF-7. With this aim, the effect of HPNEM on MCF-7 cell cycle arrest, apoptosis, and the expression of oncomiRs (miR-155 and miR-21) were evaluated. The expression of miR-155 was downregulated by HPNEM, supporting its therapeutic potential in treating breast cancer [112].

In another study, Colombo et al. (2018) created a muco-adhesive NE containing kaempferol (KPF-MNE) and examined its potential as a nasal delivery method for the rat brain after nasal injection, as well as its efficacy against a glioma cell line. Based on the results, the nasal mucosa was not harmed by the generated NEs, indicating that the nanocomposite film NE was regarded as superior to KPF-NE and free KPF in targeting the brain following intranasal delivery. The above-mentioned result was substantiated by ex vivo permeation experiments and in vivo bio-distribution investigations. Overall, the muco-adhesive NE increased apoptosis and reduced the viability of glioma cells [113].

#### 2.2.2. Flavonolignan Nanoemulsions

NEs and MEs with generally regarded as safe (GRAS) and food-acceptable components were developed for topical and oral administration to improve drug permeability, stability, and solubility [114,115,116,117,118,119].

Supersaturatable self-emulsifying drug delivery system (S-SEDDS) is designed to lessen the side effects of high surfactant levels commonly employed in SEDDS. Wei et al. [120] led a research group to develop the aforementioned formulation with plans to improve SLM oral bioavailability. The above-mentioned formulation contained a decreased concentration of surfactant combined with hydroxypropyl methylcellulose (HPMC), which was mixed with the liquid SEDDS to induce a super saturation state through preventing or minimizing SLM precipitation. In addition, the researchers investigated the solubility of SLM in several surfactants, cosurfactants, and oils at 25 °C. Labrafac^®^ CC was selected as the oil phase for S-SEDDS production due to its maximum drug solubility. Cremophor^®^ RH 40 was selected as the surfactant due to its good emulsion-forming capabilities and the reduced size of the droplets of the optimized SLM-loaded emulsion (almost 50 nm). Cremophor^®^ RH 40 was selected due to the synergistic effects of the cosurfactants Labrasol^®^ and Transcutol^®^.

Adhikari et al. [121] developed stable NEs using water as the aqueous phase, Labrafac^®^ as the oil phase, Transcutol^®^ as the cosurfactant, and Solutol^®^ HS 15 as the surfactant to deliver SLM. The radioprotective potential of the unique formulation in the aforementioned components against radiation-induced oxidative damage in normal kidney cells was studied utilizing SLM-loaded nanosuspensions. The above-mentioned formulations were created to act as SEDDS when water was used with the oil phase adding 10–15% of the solution. The vitality of HEK cells treated with various concentrations of SLM-SEDDS was reviewed and compared to drug suspensions without additives prior to radiotherapy. Radiation-induced apoptosis was estimated applying cell cycle estimates and microscopic analysis. Based on another study, the SLM-enriched NEs were designed employing various oils including castor, EVO, and sunflower [122]. SLM solubility in castor oil equaled 0.668–0.072 mg·g^−1^. However, this factor was considerably less in sunflower and EVO oils. The solubility increased to 1–2 mg·g^−1^ in the oils when Tween 80 was added to the oil (10 mg·g^−1^). The method used for preparing the aforementioned factors involved formation related to the coarse emulsions followed by exposure to high-pressure homogenization (HPH). The final mixture contained droplets with diameters ranging from 200 to 300 nm. In addition, the higher oil oxidation susceptibility led to a higher number of NEs containing the degraded drug.

Nagi et al. [123] utilized a Box–Behnken statistical design (BBD) for SLM-loaded NE optimizations applying Capryol 90 as the oil phase. The SLM was solubilized up to 40 mg·mL^−1^ based on various factors including the number of HPH technique cycles, processing pressure, and amount of surfactant/cosurfactant in the mixture. Table 2 shows some of the NE and ME structures employed to encapsulate flavonoids and flavonolignans.

### 2.3. Solid Lipid Nanoparticles

SLNs are considered as a major nanotechnology drug delivery platform [124,125]. SLNs are regarded as spherical particles with 50–1000 nm diameter, which contain a lipid matrix where the drug may be incorporated or dissolved [126]. In fact, one or more room-temperature solid lipids, surfactant, and water are involved in preparing SLNs [127]. Triglycerides, cholesterol butyrate, cholesterol, carnauba wax, beeswax, emulsifying wax, and cetyl alcohol are among the solid lipids utilized to prepare SLNs. The mechanism for lipid vesicle formation includes hydrophobic–hydrophilic interactions and the van der Waals forces of water molecules and phospholipids [68].

SLN properties including average size, drug loading capacity, release profiles, and surface charge (i.e., zeta potential) may be precisely adjusted via selecting different structures or blends of lipids, preparation method, surfactant agent(s), and aqueous or organic production media. Different targeting moieties may be applied to functionalize the above-mentioned NPs surfaces [128].

Nowadays, lipid-based carriers have reached high metabolic and physicochemical stability, along with higher biocompatibility [55,74,129,130]. Both hydrophilic and lipophilic drugs can be encapsulated in SLNPs with enhanced bioavailability and absorption, resulting in improving the biopharmaceutical profile compared to conventional colloidal carriers [131]. Emulsomes are considered as the solid-state equivalents of common multi-lamellar and unilamellar lipid vesicles in which the NPs with an internal solid fat core are surrounded by at least one phospholipid layer, resulting in making emulsomes a special type of SLN [132,133].

#### 2.3.1. Flavonoid SLNs

Rishitha assessed the effects of PTZ-induced cognitive impairment on a zebrafish model (SLN-Q) employing SLNs containing quercetin. Quercetin was found to ameliorate metabolic alterations with attenuating effect on cognition deficits which was brought on by PTZ. SLN-Q may be regarded as a potential drug in the future for treating neurodegenerative illnesses such as memory problems due to its possible anti-oxidative, anti-lipid peroxidative, and acetylcholinesterase inhibitory activities [134].

In addition, Vijayakumar conducted the physicochemical characterization and quercetin release from various SLNs, as well as creating plain and surface-coated SLNs containing quercetin. With this aim, the effect of surface coating on quercetin cellular uptake was evaluated using Caco-2 cells. Quercetin-containing SLN formulations improved the in vitro releasing profile significantly and indicated promising stability for up to 90 days of storage at 40 °C. The SLN’s surface coating was completed successfully with no compatibility issues. SLN and c-SLN displayed better quercetin uptake into Caco-2 cells than unformulated quercetin [135].

The effects of quercetin–solid lipid nanoparticles (QSLNs) on bone loss by ovariectomy were demonstrated in vivo in a different investigation. Quercetin and QSLNs suppressed the development of osteoclast cells and the expression of osteoclast-specific genes in in vitro examinations utilizing bone marrow cells treated with RANKL and M-CSF. Based on the study, oral administration of QSLNs at a dose of 5 mg/kg/day avoids endometrial hyperplastic outcomes and the loss of bone mass, bone strength, and microarchitecture brought on by estrogen deficiency. QSLNs showed substantial superior ability to stop bone loss [136].

Quercetin-enriched SLNs were created applying Arabic gum as a stabilizer and stearic acid as a core lipid employing a coacervation approach [137]. This study focuses on the synthesis and assessment of quercetin-loaded SLNs generated via coacervation. Using the aforementioned method facilitates creating SLNs containing high dose of quercetin. Based on the results, SLNs can be utilized to administer nutraceuticals like quercetin and other lipophilic medications under controlled conditions [137].

Further, Azizi et al. studied the feasibility of combining a solid lipid (palmitic acid) and an antioxidant (quercetin) into a whey-protein-isolate-stabilized SLN emulsion to encapsule fish oil. The study was conducted to find a formulation with strong lipid and antioxidant components which entrap fish oil and provide the highest physicochemical stability. The results indicated that the physical stability of the emulsions was strengthened by adding palmitic acid and reducing the size of the oil-in-water droplets. Based on the results, quercetin boosted the oxidation stability of fish oil at low amounts of palmitic acid based on thiobarbituric acid assay [138].

In another study, quercetin SLNs were prepared applying the solvent removal method with DPPC as the lipid, resulting in increasing the SLNs’ bioavailability significantly. The DPPC–quercetin binary combination improved motor nerve activity in the in vivo motor function test by 39.75% after seven days of treatment [139].

Furthermore, Liu et al. created an innovative brain-targeting drug delivery method based on OX26 antibody conjugation to PEGylated cationic SLNs (OX26-PEG-CSLN). Based on the results, OX26-PEG-CSLN could be employed as a delivery method for treating brain diseases throughout the BBB [140].

Komath et al. created SLNs (Ch-PC-SLNs) loaded with the chrysin–phospholipid complex to enhance its encapsulation. With this aim, the anti-proliferative effectiveness of the above-mentioned formulation against the MCF-7 cell line was reviewed and compared to chrysin-loaded SLNs (Ch-SLNs) [141]. Ch-PC-SLNs demonstrated zero-order release kinetics and better encapsulation efficiency than Ch-SLNs. Ch-PC-SLNs were stable after being lyophilized with mannitol as a cryoprotectant for up to three months. Ch-PC-SLNs benefit from stronger in vitro anticancer activity compared to chrysin [141].

A nanoparticulate delivery system was created to protect (-)-epigallocatechin gallate (EGCG), as the green tea main bioactive component, from deterioration during storage and digestion under simulated gastrointestinal pH conditions. EGCG-loaded SLNs were created using heat homogenization (EGCG-SLNs). Pure cocoa butter was considered as the sole lipid utilized in the production process. Mono- and diglycerides (MDG) and sodium stearoyl-2-lactylate (SSL) were combined as a surfactant. The food-grade SLNs successfully safeguarded the encapsulated EGCG throughout storage under harsh conditions and neutral pH levels, resulting in adding EGCG to food products on an industrial scale [142].

#### 2.3.2. Flavonolignan SLNs

Cengiz et al. [143] reported that SLM encapsulated in SLNs exhibited anti-hepatotoxic properties. With this aim, the formulations were applied to animals with induced hepatic damage with the co-administration of TNF-*α* and the hepatotoxin D-GaIN. The intended SLNs were prepared employing a combination of Tween 80 and SLM, and hot homogenization. The zeta potential and particle size of the SLM-loaded dispersions equaled −26.5 mV and 165–200 nm, respectively. The SLM-loaded SLNs were regarded as superior to the control while treating liver damage in both in vivo and in vitro studies. In addition, Iqbal et al. [52] extracted the organic solvent using ESE. With this aim, an organic phase containing a solid (Geleol^®^)/liquid (Sefsol^®^ 218), as an ethanolic lipid solution, and SLM was heated at 60 °C and sonicated to emulsify at 70 °C. Then, the NLC dispersions were placed into SLMs and were enhanced utilizing the Central Composite Rotatable Design (CCRD). In the next step, the optimum formulation with a particle diameter close to 126 nm and an EE of about 85% was freeze-dried and added to the Carpol hydrogel matrix to deposit epidermal tissue suited to SLM as a chemoprevention method for skin cancer. Finally, NLCs and SEDDS were applied to treat obesity-related NAFLD and enhance SLM oral bioavailability at the GIT level [52].

Further, Piazzini et al. (2018) described a procedure which involved emulsifying a lipid phase including Capryol 90 and SA in an aqueous medium containing Brij S20. Subsequent addition to cold water triggered the formation of lipid NPs. The average particle diameter, EE, and zeta potential equaled about 214 nm, 92%, and −32 mV, respectively [54]. High physical and chemical stability and increased SLM absorption were obtained in synthetic NLCs. Table 3 represents some of the SLNs and NLCs employed to encapsulate flavonoids and flavonolignans.

## 3. Clinical Studies and Market Product

As indicated, poor water solubility, low cellular uptake, low mechanical stability, and rapid metabolism of flavonoids and flavonolignans restricted their application in clinical trials. Thus, implementing carriers to deliver the aforementioned components played a critical role in potential marketed products [146]. Lipid-based nanocarriers, especially liposomes, are among the most studied carrier systems for delivering different types of therapeutic compounds. Such carriers can be considered as one of the most significant types of drug delivery systems, especially in the field of cancer therapy in various clinical trials and commercialized products [147]. There are limited marketed products and clinical trials for flavonoids and flavonolignans encapsulated in lipid-based carriers, among which liposomes and phytosomes are regarded as the most widely studied. Table 4 presents commercialized products and clinical trials of the phytochemicals-loaded lipid nanostructures.

## 4. Limitation of Translation Research to Product and Future Perspectives

Natural compounds with superior therapeutic performance have attracted a lot of attention during recent years. Recent studies apply flavonoids and flavonolignans as two groups with different therapeutic effects, which can be considered as alternatives to current drugs in cancer treatment. However, the hydrophobic nature of the above-mentioned compounds can limit their application due to relatively fast elimination in the human body. Several types of nanodrug delivery systems are proposed to overcome the above-mentioned limitation. Nanocarriers based on lipids can increase the solubility and permeability of such therapeutic compounds by their encapsulation inside their hydrophobic phase. A lot more should be learned about unique intracellular mechanisms, dosages, shelf lives, and drug interferences of the above-mentioned compounds, despite ample knowledge about them [4]. In addition, nano drug delivery systems are still in their infant stage. More in-depth studies should be conducted to completely understand toxicity, biodistribution inside human body, biodegradability, accumulation, metabolism, immunological effects, and probable side effects of the aforementioned systems on cells and genome. The market entry of nanomaterials is regarded as limited due to the lack of public acceptance. Therefore, more public education is required in this regard [150]. Lipid-based nano-formulations display low EE. Thus, a high dosage should be applied each time, leading to higher toxicity effects [146]. The other limitation is related to the high cost of precursors in some of the above-mentioned carriers, especially liposomes, which should be generated in high quantities [151]. In fact, the greater part of the present study is limited to lab-scale research in the in vitro steps and does not even reach the in vivo trials. Therefore, more studies should be conducted to overcome the barriers from bench studies to a commercialized product. SLNs exhibit limitations in drug-loading ability resulting from the high level of solid lipid core and storage stability due to their high amounts of water content and probable gelation of the dispersed phase which can affect their applications [152].

The aforementioned compounds can be applied in the future appropriately, despite the existing limitations. Lipid-based NPs can carry multiple therapeutic compounds simultaneously due to their ability to carry both hydrophobic and hydrophilic therapeutic compounds, as well as their functionalizing capabilities. This process provides the ability to treat patients in a synergistic way utilizing two or more therapeutic compounds, as well as overcoming the drug-resistant potential of some specific types of diseases, along with reducing the dosage [88,153]. More studies should be conducted to determine the potential effects of the above-mentioned components on the chemical stability and medicinal effects of the flavonoids and flavonolignans. Different therapeutic methods can be applied in addition to employing two or more therapeutic compounds. New generations of lipid NPs have been developed such as menthosomes, ethosomes, Transfersomes^®^, invasomes, flexosomes, etc., in which some structural alterations occur via adding other materials which provide a flexible membrane with deformable ability [154]. Future studies should focus on manufacturing methods and improving the amount of clinical research regarding the aforementioned compounds which can enhance their commercialization rate. This process requires a multidisciplinary approach with ethical and clinical insights [155].

## 5. Conclusions

The application of lipid-based systems for efficient and safe delivery of various compounds including naturally occurring materials has attracted a lot of attention during recent decades. Different lipid-based systems have been used to deliver flavonoids and flavonolignans such as liposomes, micro- and NEs, as well as SLNs. The above-mentioned systems can improve the stability and efficiency of their cargo in addition to the biocompatibility and biodegradability of such delivery vehicles, and can be utilized for controlled- or prolonged-delivery systems. Applying such delivery vehicles may create a pathway to clinical translation since flavonoids and flavonolignans have low water solubility and bioavailability. Many studies have been conducted on the use of the aforementioned nano therapeutic platforms for treating various diseases including cancer and hepatic ischemia, as well as diabetic nephropathy, bone loss, neurodegeneration, etc., some of which were presented in this study. The present study focused on the preparation methods for such carriers and their effects on improving the bioavailability and sustaining the release of their therapeutic agents (flavonoids and flavonolignans), and enhancing the permeability and clinical performance of the above-mentioned compounds.

The pharmacokinetic properties and non-targeted nature of flavonoids and flavonolignans is considered as the main concern of their clinical application, despite the considerable therapeutic effects in in vitro and in vivo models. The latter leads to the non-specific interaction of the aforementioned natural compounds with cells or tissues, as well as other small or macromolecules inside the body. Employing lipid delivery systems may provide the opportunity to improve their pharmacokinetic behavior, as well as targeting such systems to the precise site of action via different targeting strategies. Thus, using lipid-delivery systems for flavonoids and flavonolignans is not regarded as the last step in proposing the above-mentioned naturally occurring compounds as therapeutic agents. Bioengineering of lipid-based delivery systems is considered as a prerequisite in clinical translation. Further clinical trials should evaluate the aforementioned formulations extensively in the human body.

## Figures and Tables

**Table 1:** Liposomal structures employed to load flavonoids and flavonolignans.

Phytochemical Type	Cargo	Carrier	Disease	In Vitro/In Vivo	Major Outcomes	Ref.
Flavonoid	Quercetin	Folic-acid-modified liposome	Osteosarcoma (OS)	In Vitro	The results indicated a new quercetin-based anti-OS treatment via the JAK2-STAT3-PD-L1 signaling axis.	[82]
Flavonoid	Quercetin	PEGylated liposomes	Diabetes mellitus (DM)	In Vivo	Attenuating AGE expression, mitigating oxidative stress, and arresting DN development in STZ-induced DN rats.	[83]
Flavonoid	Quercetin	Liposomes	IRI	In Vitro/In Vivo	The results indicated that quercetin liposomes could be a useful instrument for resolving therapeutic bottlenecks in hepatic IRI.	[84]
Flavonoid	Quercetin	PEGylated liposomes	Cervical carcinoma	In Vitro/In Vivo	Based on the results, PEG-Que-NLs exhibited promising application prospects in treating malignant tumors due to their ability to target tumors, gradual release, and improved quercetin solubility.	[85]
Flavonoid	Quercetin	Liposomes	Allergic effects in RBL-2H3 cells	In Vitro	The results indicated that quercetin liposomes may reduce histamine calcium influx and hexosaminidase release significantly.	[86]
Flavonoid	Quercetin	Liposomes	Liver neoplasms	In Vivo	Suppressed excessive angiogenesis and residual tumor growth reduce metastasis and increase experimental lifespan among animals.	[87]
Flavonoid	Fisetin	PEGylated liposomes	GBM	In Vitro	The liposomal formulation contains both medications with a diameter of 173 8 nm (PDI = 0.12 0.01), as well as a drug loading of fisetin and cisplatin of 1.7 0.3 and 0.8 0.1%, respectively. In addition, the liposomal formulation contains DOPC/cholesterol/DODA-GLY-PEG2000 at a molar ratio of 75.3/20.8/3.9.	[88]
Flavonoid	Luteolin	Elastic liposomes	Breast cancer	In Vitro/Ex Vivo	The results indicated that Span 80 was considered as the best edge activator among the studied surfactants (Span 60 and Brij), which is related to negative glass transition temperature.	[89]
Flavonoid	Chrysin	Liposomes	Breast cancer	In Silico/In Vivo	Based on the current study, chitosan may shield and encase chrysin, resulting in increasing its cytotoxicity and bioavailability.	[90]
Flavonoid	Chrysin	Liposomes	HIR	In Vitro	Chrysin biocompatibility is enhanced by the liposomal drug delivery technology, and LC may lessen liver I/R toxicity and be a useful medication for treating and preventing HIR-induced injury.	[91]
Flavonoid	Genistein and plumbagin	PSMA-specific antibody conjugated liposomes	Prostate cancer cells	In Vitro	The created liposomal formulations exhibit long-term (several weeks) stability in PBS and water, and a hydrodynamic diameter of between 80 and 100 nm.	[92]
Flavonolignan	Silibinin	Liposomes	Liver neoplasms	In Vitro	The results indicated that cholesterol affects drug-entrapment and drug-leakage properties significantly. About 90% of the medication was loaded into such vesicles.	[50]
Flavonolignan	SLM	Liposomes	Improved the SLM bioavailability	In Vitro	This formulation which employs safe components and a buccal spray liposomal dose form can meet the SLM needs of children with liver conditions.	[72]
Flavonolignan	SLM	Liposomes	Cancer (pharmacokinetics evaluation)	In Vivo	SLM liposomes could encapsulate materials at a rate of over 90% with an average particle size of roughly 238.8 nm. Oral administration of SLM proliposomes leads to their high bioavailability.	[98]
Flavonolignan	SLM	Liposomes containing a bile salt	Chronic liver, cirrhosis and hepatocellular carcinoma (pharmacokinetics evaluation)	In Vitro/In Vivo	SM-Lip-SEDS indicated enhanced in vitro drug release compared to SM powder.	[95]
Flavonolignan	SLM	Bilosomes	Hepatoprotective	In Vitro/In Vivo	The results indicated the possible application of bilosomes for enhancing SM hepatoprotective effect when administered orally.	[96]
Flavonolignan	SLM	Glyceryl monooleate/poloxamer 407 liquid crystalline matrices	Improving the bioavailability of SLM	In Vitro/In Vivo	Pharmacokinetic tests revealed 3.5 times more SLM oral bioavailability compared to Legalon^®^, a commercial SLM formulation, and considerably higher peak concentrations with SLM GMO/P407 LCM.	[43]
Flavonolignan	SLM	Organogel with pluronic-lecithin (PLO)	Atopic dermatitis	In Vitro/Ex Vivo	A unique topical formulation of SLM which is loaded with PLO gel might be launched.	[97]

**Table 2:** Nanoemulsion and microemulsion structures employed to encapsulate flavonoids and flavonolignans.

Phytochemical Type	Cargo	Carrier	Disease	In Vitro/In Vivo	Major Outcomes	Ref.
Flavonoid	Naringenin	NEs	Alzheimer disease (AD)	In Vitro	The naringenin-loaded NE alleviated the direct neurotoxic consequences of Aβ on SH-SY5Y cells significantly. This process was once related to a downregulation of APP and BACE expression, indicating decreased amyloidogenesis.	[104]
Flavonoid	Daidzein	NEs and NEGs	Melanoma	In Vitro	All of the formulations benefitted from appropriate pH values for pores and skin complying with zero-order launch kinetics. The DZ launch fees acquired for DZ-NE and DZ-NEG equaled 2.701 ± 0.265 and 1.325 ± 0.117 μg/cm^2^/h, respectively.	[105]
Flavonoid	Quercetin	NEs	Heart failure and vascular disease (CVD)	In Vitro/In Vivo	Based on the results, quercetin and other polyphenolic substances are enhanced by therapeutic NE.	[106]
Flavonoid	Quercetin	NEs	Diabetes mellitus	In Vivo	The optimized quercetin NE exhibited appropriate stability for forty-five days. The Que-NE demonstrated most useful oral bioavailability unlike Que-PD.	[107]
Flavonoid	Quercetin and cisplatin	NEs	Human breast carcinoma	In Vitro	The new formulations multiplied the antitumor recreation of each of the molecules and the synergistic impact of the cisplatin/quercetin unlike the MDA-MB-231.	[108]
Flavonoid	Quercetin	NEs	High cholesterol	In Vitro/In Vivo	Based on the results, hepatic bile acid synthesis and fecal cholesterol excretion play at least a minimal role in quercetin preventive effects on lipid abnormalities.	[109]
Flavonoid	Quercetin	Olive oil NEs	Cancer	In Vitro	Quercetin-entrapped NEs formed stable complexes with HSA and HTF through improving hydrophilic–hydrophobic interactions compared to the non-entrapped quercetin.	[110]
Flavonoid	Quercetin and vincristine	NEs	Leukemia	In Silico/In Vitro	Direct inhibition of ABCB1 efflux activity by the unloaded NE demonstrated the structure–activity link.	[111]
Flavonoid	Hesperidin	NEs	Cancer	In Vitro	HPNEM reduced mir-21 and mir-155 expression, demonstrating its therapeutic potential for breast cancer.	[112]
Flavonoid	Kaempferol	Muco-adhesive NE	Glioma cell line	In Vitro/Ex Vivo/In Vivo	KPF from KPF-MNE displayed higher permeation across the mucosa in ex vivo diffusion studies.	[113]
Flavonolignan	SLM	NEs	Stabilization of SM-NE droplets	In Vitro	Based on the studies, tea saponin has the capacity to prepare SM-NE as natural emulsifiers and act as cryoprotectants to lessen SM-NE damage during freeze-drying.	[119]
Flavonolignan	SLM	MEs	Evaluated the drug delivery systems	In Vitro	The studies indicated prolonged release for MEs compared to SLM solution.	[117]
Flavonolignan	SLM		Improving the bioavailability of SLM	In Vitro	In vitro release of SLM from SMEDDS determined by a dialysis method and an ultrafiltration method showed first-order kinetics and was typical of sustained characteristics.	[118]
Flavonolignan	SLM	MEs	Improving the bioavailability of SLM	In Vitro/In Vivo	SMEDDS is considered as an effective oral drug delivery system for non-water-soluble medications. The most useful system with the exceptional self-microemulsifying and solubilization potential contained 10% (*w/w*) of ethyl linoleate, 30% of Cremophor EL, and 60% of ethyl alcohol.	[116]
Flavonolignan	Silybin	MEs	Improving the bioavailability of SLM	In Vitro/In Vivo	This study indicates the effectiveness of hyper-saturable formulations as a delivery method for improving the oral bioavailability of drugs which are not regarded as highly soluble.	[120]
Flavonolignan	SLM	NEs	Hepatic diseases	In Vitro	The nanosized formulation allows enhancing bioavailability inside body.	[121]
Flavonolignan	SLM	NEs	A pharmacokinetic study	In Vitro/In Vivo	This study indicates that NE technology can enhance SLM biopharmaceutical capabilities.	[25]
Flavonolignan	SLM	NEs	Hepatic	In Vitro/In Vivo	The drug dissolved rapidly from the NPs, reaching almost 80% during 15 min, indicating three-fold higher dissolution than that of the commercial product.	[26]
Flavonolignan	SLM	NEs	Analyzing bio-accessibility and -stability	In Vitro	Castor oil deteriorated silybin with less stability than extra virgin olive or sunflower oil.	[122]
Flavonolignan	SLM	NEs	Pharmacokinetic study	In Vitro	Based on a pharmacokinetic study, SLM in NE exhibited a significantly (P 0.05) better oral bioavailability than that in oral solution, indicating that NE may be an appropriate oral delivery method for SLM.	[123]

**Table 3:** SLNs used to encapsulate flavonoids and flavonolignans.

Phytochemical Type	Cargo	Carrier	Disease	In Vitro/In Vivo	Major Outcomes	Ref.
Flavonoid	Apigenin	SLNs	Diabetic nephropathy	In Vitro/In Vivo	The encapsulation efficiency of pigenin–SLNP equaled 78.90%. A medicine releases on average 71.52% of its dose every 24 h with a fast release of 34.28% every five hours.	[144]
Flavonoid	Quercetin	SLNs	Neurodegenerative disorders	In Vitro	Employing as a therapy for neurodegenerative illnesses like memory impairments.	[134]
Flavonoid	Quercetin	SLNs	Carcinoma (Caco-2 cell line)	In Vitro	Enhancing Caco-2 cells’ absorption of quercetin by SLN and more quickly than pure quercetin; quercetin from SLN dispersion was released.	[135]
Flavonoid	Quercetin	SLNs	Post-menopausal osteoporosis	In Vitro/In Vivo	A notable distinction in the release pattern of QSLNs and quercetin. A continuous release was observed in the case of the QSLNs and 45 6.33% of the medication was released by the end of 12 h unlike a release of less than 10% for pure quercetin.	[136]
Flavonoid	Quercetin	SLNs. Stearic acid as a core lipid and arabic gum as a stabilizer	Improving the bioavailability of SLM	In Vitro	The system displayed a regulated antioxidant impact compared to free quercetin, indicating that the encapsulated nutraceutical retains a significant amount of its antioxidant activity (around 81% of that of free quercetin).	[137]
Flavonoid	Quercetin	SLNs stabilized by whey protein isolate	Studying the physicochemical properties	In Vitro	Quercetin improved fish oil oxidative stability at low palmitic acid concentrations significantly.	[138]
Flavonoid	Quercetin	SLNs	Spinal injuries	In Vitro/In Vivo	Quercetin only displayed a 62.20 1.06% drug release at the end of the 12-h dissolving research. However, its lipid SLNs showed an 84.99 1.42% drug release. The bioavailability enhanced significantly with the SLNs.	[139]
Flavonoid	Baicalin	PEGylated cationic SLNs conjugated with OX26 antibody	Brain disorders	In Vitro	OX26-PEG-CSLN is among possible delivery strategies for treating brain diseases throughout the BBB.	[140]
Flavonoid	Chrysin–phospholipid	SLNs	Cancer	In Vitro	Ch-PC-SLNs demonstrated zero-order release kinetics and a better encapsulation efficiency than Ch-SLNs.	[141]
Flavonoid	(─)-epigallocatechin-3-gallate (EGCG)	SLNs	For food applications	In Vitro	This technique is considered as efficient for incorporating EGCG on a wide scale into food items.	[142]
Flavonolignan	Silibinin and d-α-tocopheryl polyethylene glycol 1000 succinate (TPGS)	SLNs	Breast cancer	In Vitro/In Vivo	The optimized SLNs exhibited an appropriate serum stability with around 45 nm in size entering MDA-MB-231 breast cancer cells effectively.	[130]
Flavonolignan	SLM	Lipid NPs	Studying the drug delivery system	In Vitro	Improved solubility, alternative routes including improved absorption and lymphatic transport, or perhaps both can increase bioavailability.	[129]
Flavonolignan	SLM	SLNs	Study of the drug delivery system	In Vitro	Based on the studies, cold-SM-SLNs can increase SM oral bioavailability, indicating a potential use for SM oral drug delivery system to the liver.	[131]
Flavonolignan	SLM	SLNs	Diverse liver and gallbladder disorders	In Vitro/In Vivo	SM-loaded SLN might be a beneficial approach for distributing poorly water-soluble Sm beyond offering favorable hepatic protection.	[143]
Flavonolignan	SLM	NLCs	Studying the drug delivery system	In Vitro	Possibility of controlled drug release and targeting, lack of carrier biotoxicity and biodegradation, and absence of manufacturing scale-up difficulties in the food and pharmaceutical sectors.	[54]
Flavonolignan	Silybin	Mixed micelles	Studying the drug delivery system	In Vivo	Based on the studies, bioavailability increased by 7–9 times compared to a fast-release silybin solid dispersion formulation.	[55]
Flavonolignan	Silybin	NLCs	Biodistribution and pharmacokinetic studies	In Vitro	The results indicate that silybin continuous release and targeting through NLC is a possibility.	[53]
Flavonolignan	SLM	NLCs	Analyzing epidermal drug deposition enhancement	In Vitro	The results indicate the capability of NLC gel to deliver SLM topically.	[52]
Flavonolignan	Silybin	Lipid nanospheres	Study of pharmacokinetics	In Vitro/In Vivo	Unlike SLM, SLM proliposome is regarded as a stable and easy-to-make formulation which supports SLM gastrointestinal absorption.	[145]

**Table 4:** Some of the commercialized products and clinical trials of the phytochemicals-loaded lipid nanostructures [148,149].

Product Name	Drug	Nanostructure	Application	Clinical Trials
Gallic acid phytosome^®^	Gallic acid	Phytosome	Antimicrobial and anti-apoptotic activities	NM ^1^
Ginkgoselect^®^ phytosome^®^	Ginkgoselect	Phytosome	Antioxidant activity and hepatoprotective feature	NM
Silybin phytosome^®^	Silybin	Phytosome	Hepatoprotective and antioxidant abilities	NM
Ginseng phytosome^®^	Ginseng	Phytosome	Nutraceutical compound and immunomodulator agent	NM
Green tea phytosome^®^	Green tea	Phytosome	Antioxidant and anticancer ability	NM
Grape seed phytosome^®^	Grape seed	Phytosome	Antioxidant and cardioprotective ability	NM
Hawthorn phytosome^®^	Hawthorn	Phytosome	Cardioprotective agent	NM
Quercetin phytosome^®^	Quercetin	Phytosome	Antioxidant and anticancer ability	NM
Naringenin phytosomes^®^	Naringenin	Phytosomes	Antioxidant ability	NM
Dasatinib + quercetin phytosome	Quercetin	Phytosomes	Antioxidant	2 (NCT04313634)

^1^ NM: not mentioned.

## Data Availability

Not applicable.
